# Aortic Size Index Versus Aortic Diameter in the Prediction of Rupture in Women With Abdominal Aortic Aneurysm

**DOI:** 10.7759/cureus.58673

**Published:** 2024-04-21

**Authors:** John O Olukorode, Chidera N Onwuzo, Emmanuel O Otabor, Nwachukwu O Nwachukwu, Raymond Omiko, Olutomiwa Omokore, Heritage Kristilere, Yetunde Oladipupo, Rolake Akin-Adewale, Oluwatosin Kuku, Joshua O Ugboke, Thummim Joseph-Erameh

**Affiliations:** 1 Internal Medicine, Benjamin S. Carson College of Health and Medical Sciences, Babcock University, Ilishan-Remo, NGA; 2 Internal Medicine, University Hospital Coventry and Warwickshire, Coventry, GBR; 3 Surgery, College of Medicine, University of Ibadan, Ibadan, NGA; 4 Internal Medicine, College of Health Sciences, University of Ilorin, Ilorin, NGA; 5 Internal Medicine, Obafemi Awolowo College of Health Sciences, Olabisi Onabanjo University, Ogun, NGA; 6 Internal Medicine, College of Medicine, University of Lagos, Lagos, NGA

**Keywords:** aortic aneurysm, rupture in women, aaa rupture, aortic diameter, abdominal aortic aneurysm, aortic size index

## Abstract

Abdominal aortic aneurysms (AAAs) pose significant challenges in clinical management, particularly in female patients, whose unique anatomical and physiological characteristics influence rupture risk. While aortic diameter (AD) has traditionally been the primary metric for predicting rupture, its limitations, especially in women, have spurred exploration into alternative measures such as the aortic size index (ASI). This review examines the anatomy and physiology of AAAs in women, gender-specific challenges in diagnosis and management, and the comparative effectiveness of ASI versus AD in predicting rupture risk. ASI, calculated as AD divided by body surface area (BSA), offers a more nuanced assessment by adjusting for individual body size differences, potentially mitigating gender disparities in rupture rates. Comparative analyses indicate ASI's superiority in predicting adverse aortic events, particularly in women, thereby advocating for its integration into clinical practice to improve patient outcomes. Additionally, emerging techniques such as 3D volumetric measurements and biomechanical assessments show promise in enhancing rupture risk prediction, heralding a shift toward more personalized and effective management strategies for AAA patients.

## Introduction and background

Abdominal aortic aneurysms (AAAs) have long been recognized as a critical cardiovascular condition primarily affecting the elderly male population. However, the incidence of AAAs in female patients has gained increasing attention in recent years. Traditionally considered a male-dominated disease, the prevalence of AAAs in women presents a unique set of challenges, from diagnosis to management.

The rupture of an AAA is a catastrophic event associated with high mortality rates, making early identification and intervention crucial for improving patient outcomes. In female patients, the challenges lie not only in the less frequent occurrence of AAAs but also in the potential for differing anatomical and physiological factors influencing the rupture risk. Establishing accurate predictive measures tailored to the female population is paramount for enhancing clinical decision-making and ultimately saving lives.

Current predictive measures for AAA rupture predominantly revolve around assessing aortic diameter (AD). However, this approach has inherent limitations, especially when applied to female patients. The current indications for surgical intervention for AAA in men include AAA diameter greater than 5.5 cm measured through ultrasonography, symptomatic AAA, and rapidly expanding AAA (more than 1 cm/year) irrespective of the absolute diameter [1]. The traditional threshold for intervention may not be as applicable due to variations in aortic size, structure, and biomechanics in women. As a result, researchers have sought alternative methods, and one promising avenue is the exploration of the aortic aneurysm size index - a metric that considers both diameter and other anatomical factors. Despite these advancements, challenges persist in accurately predicting rupture, and understanding the limitations of existing measures is crucial for paving the way toward more effective and gender-specific predictive models.

This review will explore the AD and aortic size index (ASI) as metrics for predicting AAA rupture risk in women, as well as their clinical implications and limitations. Measurements for AD in the studies reviewed in the article were conducted via ultrasonography.

## Review

Anatomy and physiology of AAAS in women

Although the prevalence of AAAs is three to five times higher in men, the disease progression follows a more rapid course in women, introducing gender-specific differences that shape the disease's manifestation [2,3]. Anatomically, women present unique considerations in the context of AAAs. Female aortas tend to be smaller in diameter compared to male aortas. A study by Tran et al. found an average luminal diameter of 8.8 mm compared to 11.8 mm in men (P<0.001) at the common iliac artery [4]. The female anatomy is implicated in the hemodynamics of the abdominal aorta. Diastolic flow reversal in the infra-renal aorta has been found to be lower in women than in men [5]. This has been attributed to the low-resistance vasculature of the uterus, smaller vessel size, and more compliant aortas in women [5,6]. Diastolic flow reversal leads to oscillatory blood flow patterns, causing increased stress on the aortic endothelium. This has pro-inflammatory effects and has been implicated in the initiation of aneurysms, hence, the lower prevalence of AAAs in women. The smaller dimensions and increased compliance of the aorta in women affect the biomechanics of the arterial wall, influencing the propensity for aneurysm rupture. Wilson et al. in their study concluded that the female gender has a shorter time to AAA rupture (hazards ratio (HR), 2.78; 95% CI, 1.23 to 6.28; P=0.014) [7].

Hormonal influences represent a key facet of the anatomical and physiological disparities in AAAs between men and women. Estrogen, a hormone predominant in females, has been implicated in conferring protective effects on the cardiovascular system. In mice, estradiol was shown to attenuate the effects of angiotensin II in the development of aneurysms. Angiotensin II has been shown to have inflammatory effects on the aorta, mediating atherosclerosis. A total of 42% of mice that received angiotensin II and estradiol developed aneurysms as opposed to 90% that received angiotensin II only. It was also observed that estradiol decreased the expressions of intracellular adhesion molecule-1, vascular cellular adhesion molecule-1, E-selectin, monocyte chemotactic protein-1, and macrophage-colony-stimulating factors in the aorta [8].

Studies suggest that premenopausal women, who experience higher levels of estrogen, may exhibit a reduced incidence of AAAs. Conversely, the postmenopausal phase, characterized by a decline in estrogen levels, is associated with an increased risk of AAAs in women. A case-control study showed that the mean age at menopause of women who developed AAA was 47.7 compared to 49.9 in those who did not [9]. The constellation of these anatomic and physiologic features in women is protective of AAA, explaining why prevalence is very low, and also why there is no place for routine surveillance. This may pose a challenge in the identification, presentation, and progression of AAA in female patients.

Gender-specific challenges and considerations

The prevalence of AAA is about six times greater in men than in women when an ultrasound (US)-measured maximum diameter of ≥3 cm is used [10]. This low prevalence in women has been attributed to the protective effect of estrogen and other anatomical factors as discussed. The median age at AAA management is higher in women. A study revealed that women undergoing intact repair of an aneurysm were three years older than men (median age 75 vs. 72 years, P<.001), while those undergoing ruptured repair were five years older (median age 78 vs. 73 years, P<0.001) [3]. A similar study showed the average age of women presenting with rupture to be 80 years compared to 75 years in men [11]. 

Time to rupture has been found to be shorter in women when adjusted for age and diastolic blood pressure (which was also found to decrease the time to rupture). This was attributed to smaller diameters and increased distensibility of their abdominal aorta [7,12]. In a retrospective study conducted by Brown et al., which involved 476 patients with an AAA diameter of 5.0 cm to 5.9 cm considered unfit for surgery, the average risk of rupture was 3.9% in female patients compared to 1.0% in their male counterparts [13]. This suggests a lower threshold for rupture and the need for surgery at lower AAA diameters in women. 

Studies have shown that the hospitalization rate for women with AAA is lower than for men. Men were 4.8 times more likely to be hospitalized for an intact AAA and five times for ruptured AAA than women [14]. Women are also less likely to undergo surgical intervention for AAA. In one study, 22.4% of women versus 34.3% of men underwent aneurysm repair for intact AAA. Similar findings were observed for ruptured AAA (49.1% vs. 61.3%) [14]. This is consistent with findings by Semmens et al., in which 37% of women with ruptured AAA compared to 63% of men underwent operation [11]. The reasons for this are not known, but it has been attributed to the general lesser aggressiveness with respect to the treatment of cardiovascular diseases, for example, coronary heart disease in women. Also, obesity, which is a common comorbidity, has been suggested as a possible factor contributing to the lower surgical intervention rate in women [14]. Furthermore, due to the reduced iliac artery size and shorter, more angulated aortic neck, EVAR (endovascular aneurysm repair) was shown to have reduced applicability in women [15].

In-hospital mortality rates and postoperative complications are higher in women. Women have a 45% greater risk of dying than men after repair of ruptured AAA [14]. McPhee et al. recorded a similar mortality rate after repair of ruptured AAA (43% vs. 36% in women). For repair of intact AAA, in-hospital mortality rates were also higher in women for both endovascular (2.1% vs. 0.83%, P<0.0001) and open repairs (6.1% vs. 4.0%, P<0.0001) [16]. This finding is consistent with many other studies [17,18,19,20]. Postoperative complications including transfusion, pulmonary complications, and bowel ischemia are more common in women after OAR and EVAR. Arterial injury, limb ischemia, renal, and cardiac complications are more common in women after EVAR [21]. The reason for the observed difference in mortality outcomes is not clear. It has been attributed to a higher age at presentation in women. The higher rates of comorbid conditions, seen as a risk factor for other cardiovascular diseases, may also explain this difference. For instance, a high proportion of women who developed AAA have a smoking history (OR=3.29, 95% CI 1.86 to 5.80, P<0.0001) [22]. Katz et al. attributed this difference to a delay in referral due to reliance on AAA diameter as an indicator for surgical intervention in women [14]. For a particular AAA diameter, women have more advanced disease than men. Hence, the need for a more accurate indicator that takes the female anatomy into consideration. Deery et al. noted that after adjusting for ASI rather than for AD, the association between female sex and mortality reduced [23].

AD: a traditional metric for rupture risk

Overview of Using AD as a Predictor

**Figure 1:**
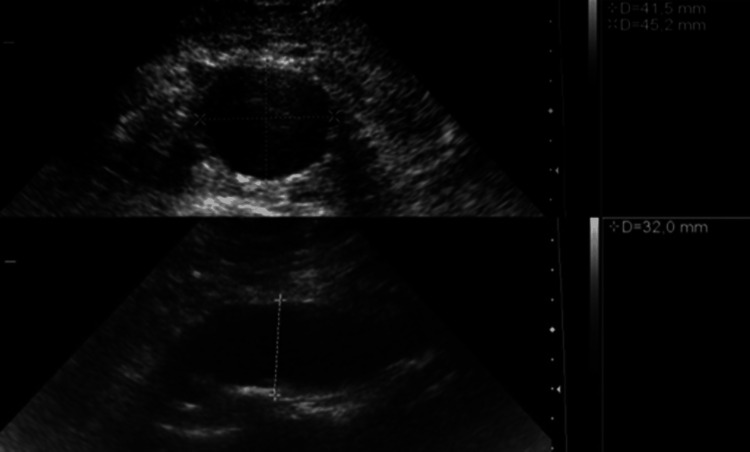
US Measurements of AD in Transverse (Top) and Longitudinal (Bottom) Planes. AD, aortic diameter; US, ultrasound

It has been well established that the risk of AAA rupture increases with increasing size. From the onset, AD has been used as a diagnostic and predictive value for AAA and its rupture risk. AAA is defined as an AD greater than 3 cm measured using a transabdominal US [24]. Measurements are made from the outer wall to the outer wall of the perpendicular aortic axis as seen in Figure 1 [25].

Aneurysms with a diameter greater than 6 cm have a 25% annual risk of rupture according to the British Society of Interventional Radiology, while the Society for Vascular Surgery (SVS) puts it at 20% [24,26]. AAA is a common cause of a ruptured aorta, another being trauma. It is important for clinicians to be able to monitor the risk of rupture as this informs the decision of when surgical interventions are required. The SVS recommends surveillance imaging annually for diameters of 4.0 to 4.9 cm and elective repair for patients with a fusiform aneurysm of 5.5 cm or more, with a one-time US screening in all men and women aged 65 to 75 who have a history of tobacco use; men with a family history of aneurysm aged 55 and above; and women who have a family history and are aged 65 and above [24].

The principle of using AD is based on Laplace’s hydraulic principle [27]. This states that wall tension is directly proportional to the product of the intraluminal pressure and the radius. The wall tension is also inversely proportional to the thickness of the wall. As an aneurysm enlarges, wall thickness reduces (with reduced elastin concentration), leading to increased wall tension and subsequent rupture [28].

*Historical Context and Evolution of AD* *Assessment*

The use of AD as a predictor for rupture was informed by a retrospective review of 24000 autopsies conducted over a period of 23 years, from 1952 to 1975, wherein 40% of AAAs with a diameter greater than 5 cm were ruptured [29]. This corroborated the work of Szilagyi et al. in 1972, where they studied the outcomes of patients over a timeline of 19 years who did not have surgical repair for AAA. Forty percent of them had a rupture within the first year of diagnosis, with 42.5% of those 40% having a diameter greater than 6 cm [30].

Ultrasonography has been the most popular investigation for a suspected AAA and for surveillance. It detects the presence of an aneurysm, the size (diameter), and the extent of the aneurysm. It has a high sensitivity above 95% and specificity above 99% and is cost-effective [31]. Computed tomography angiography (CTA) is the gold standard for preoperative examination of an AAA [32].

Limitations and Challenges Associated With Relying Solely on AD

In the study conducted by Darling et al. in 1977, 40% of AAAs with a diameter greater than 5 cm were ruptured, which stands to reason. The conundrum found was that 40% of the AAAs with diameters of 7 to 10 cm were not ruptured, and diameters less than 5 cm were ruptured in 13% of the autopsies conducted [29]. This has since led to the search for more sensitive predictors of rupture, which include the expansion rate, wall stiffness, peak wall stress, wall tension, and peak wall rupture index (a function of both peak wall stress and residual wall strength) [33].

Accurate measurement using AD is limited by the tortuousness of the aorta, morphological variations like saccular aneurysms, and observer variability [27]. Variability can occur when identifying the site of the maximum diameter, the orientation of the diameter (either sagittal or coronal), the axial plane chosen, or whether the wall thickness is included in the measurement of the diameter [34]. The imaging modality with the highest risk of variability is ultrasonography.

Moreover, AD alone does not account for gender differences, given that men and women have different body surface areas (BSA) and thus have different risks of developing ruptures. It is known that women have a risk four times higher than men in the development of a rupture at any given diameter; therefore, AD alone is not a significant predictor of rupture in individual patients [35].

This has thus led to the need to factor in the difference in BSA among men and women, which is seen in the ASI, dividing AD by BSA. The threshold for ASI for the diagnosis of AAA is ≥1.5. Male prevalence of AAA using AD and ASI was found to be the same at 5.7%, but it differed significantly in females, 2.4% using AD and 4.5% using ASI, almost double the value [36].

The use of AD as a predictive value for AAA rupture has led to the absence of routine screening for women due to the lower prevalence it provides [37]. However, the use of ASI for prediction will show the need to offer screening to women, knowing that they have a higher chance of rupture at any diameter, and they suffer more cardiovascular consequences.

ASI: concept and calculation

The ASI is defined as the AD divided by the BSA. ASI is calculated by the following formula: ASI=AA diameter (cm)/BSA (m2) [38,39]. The concept of ASI is such that it adjusts AD to BSA rather than using the same cut-off limit for people of different BSAs [40]. An increase in ASI has been associated with an increased risk of AAA rupture, particularly among women [39]. 

AAAs are known to have poorer outcomes in females compared to males. The reasons for this observation have been postulated to be multifaceted, including older age, challenging anatomy, smaller caliber vessels, and undiagnosed cardiovascular diseases. Another interesting postulation suggests that because women are generally smaller in size than men, aortic dilatation of certain sizes is considered relatively larger compared to men, indicating more advanced disease at the time of presentation and treatment [35]. Consequently, the joint council of the American Association of Vascular Surgery and the Society of Vascular Surgery advises a lower cut-off for AAA repair in women [35].

By employing BSA, it was shown that factoring in body size mitigated the gender disparity in rupture rates. This supports the long-held suspicion that the higher occurrence of ruptures in women with smaller ADs is attributed to their aneurysms being proportionally larger in relation to their body size when compared to men. It is crucial to emphasize that BSA and ASI consider body size and are not mere indicators of obesity [35].

Comparative Analysis of ASI and AD

AAA predominantly affects elderly males [41]. However, recent evidence suggests that its prevalence may be influenced by how AAA is defined [35]. Traditionally, AD has been used not only to establish the presence of AAA but also as a key predictor of rupture risk [13]. Evidence shows variations in aortic size between genders, prompting exploration into whether alternative metrics such as the ASI might offer a more accurate assessment [42].

The landmark study that first proposed the ASI conducted a comparative analysis to assess its predictive abilities alongside those of AD in anticipating adverse aortic events [43]. Their findings demonstrated the superiority of ASI in predicting rupture, dissection, and mortality compared to maximal AD alone. While both initial aortic size/diameter and ASI were linked to an elevated risk of aortic rupture, ASI showed a stronger association. Patients were stratified into three risk groups based on ASI: less than 2.75 cm/m^2 ^for low risk (around 4% per year), 2.75 to 4.24 cm/m^2^ for moderate risk (about 8% per year), and above 4.25 cm/m^2^ for high risk (approximately 20% per year). Increasing ASI was also associated with a higher incidence of dissection, though to a lesser extent than rupture.

In a study by Jones et al. involving 4115 individuals, AAA prevalence was examined across screening groups, focusing on correcting for BSA, that is, ASI [36]. Significant differences in AAA prevalence between genders were observed based on absolute aortic size thresholds but not with ASI, suggesting the influence of BSA variations. For instance, while males exhibited a 5.7% prevalence with a 30 mm AD threshold when measured via ultrasonography, females showed a lower 2.4% prevalence. Interestingly, using ASI ≥1.5, male prevalence remained consistent, whereas female prevalence substantially increased to 4.5%, indicating a notable rise in cases below the 30 mm threshold. The study suggests ASI may offer a more accurate depiction of AAA prevalence, potentially reducing diagnostic errors and promoting equitable screening strategies.

A study in Norway affirmed ASI’s role as a predictor of AAA and aortic growth for both genders [38]. It identified age, smoking, male gender, higher BMI, and high total cholesterol as linked factors to AAA development. When ASI was factored into the analysis, age's predictive significance diminished, while it retained its predictive value when considering AD. However, in contrast to a previous study, where ASI exhibited better predictive capability for rupture than AD specifically among women, this study did not find significant variation in the association between ASI and AAA in men and women; AD was found to be a significantly better predictor of aortic growth than ASI [44].

In a separate study examining sex-based disparities in mortality and morbidity following intact AAA repair, it was observed that women experienced higher rates of mortality and major complications post-EVAR and open repair [23]. Even after accounting for age, comorbid conditions, and aneurysm diameter, female sex remained linked to increased mortality and major complications post-EVAR. However, a notable finding was that women generally presented with smaller AAA diameters but larger ASI at the time of repair. Upon adjusting for ASI, women no longer exhibited elevated risks of mortality or morbidity, suggesting a potential delay in intact repair for women compared to men. These findings were supported by another study, where it was noted that in comparison to men, women had a US-measured mean aneurysm diameter that was 2 mm smaller for intact aneurysms and 7 mm smaller for ruptured ones [35]. Additionally, a significantly higher proportion of women (43% vs. 36%) underwent repair of intact aneurysms at diameters <5.5 cm and <5.0 cm (13% vs. 11%).

Lo et al. conducted a retrospective analysis of patients from the Vascular Study Group of New England (VSGNE) database who underwent either endovascular or open repair for AAA [35]. They calculated various body size indices, such as height, weight, and BSA, relative to aneurysm diameter. These indices, along with other relevant clinical variables, were utilized to develop separate age-adjusted and multivariable-adjusted logistic regression models for men and women. The study found that in men, AD was the most significant predictor of ruptured repair, whereas in women, ASI emerged as the strongest predictor. Interestingly, after adjusting for ASI and other factors, aneurysm diameter alone did not significantly predict ruptured repair in women.

Incorporating body size through BSA and ASI can help address the discrepancy in rupture rates between genders, highlighting the significance of factoring in these variables when making decisions regarding AAA management.

Challenges and Controversies in Implementing ASI as a Standard Predictor

Several reports have discussed the superiority of ASI as a predictor of future rupture in women with AAA over the more conventional AD. The Joint Council of the American Association of Vascular Surgery implemented a higher-diameter baseline for surgical intervention in women [45]. 17% of women underwent surgical repair for aneurysms ruptured at <5.5 cm [35]. BSA and ASI analysis introduced the idea that female patients with AAA ruptures happen at smaller ADs. This reinforces ASI as a better predictor in women due to greater variations in body size and introduces a threshold of ≥2.5cm/m^2^ for early repair [35]. Re-calibrating for ASI rather than AD in multivariable research also reduces sex disparity outcomes [46]. This index, as an informative and decision-making tool, should be incorporated into clinical practice [46]. Varying use of different ASI baselines and thresholds results in surgery avoidance rates ranging from 42% to 83%. This can serve as a basis upon which the use of ASI as a standard predictor for rupture in AAA in women is institutionalized [46]. Additionally, the ability to use it as a surveillance tool in individualized cases also supports the viewpoint discussed. Various results suggest that ASI may be a more useful screening tool as well as for determining the indications for aneurysm repairs following further research and studies [47].

A population-based prospective study describes ASI as an important and independent predictor of incidence AAA as well as a predictor of aortic growth of >5 mm over seven years in women [38]. Furthermore, in a population-based cohort study of ruptured AAAs in women, an increase in the supra-renal ASI was identified as a potential marker for future rupture. This necessitates the surveillance of patients with ASI, therefore, increasing attention and potentially benefiting from early intervention repair [48]. It is important to note that there are varying factors associated with the development and rupture of AAA, and a direct comparison between AD and ASI as a standard predictor is not straightforward. ASI assists in identifying female patients in whom early repair of aneurysms would be highly beneficial considering the absence of a clear, universally defined threshold.

Areas of uncertainty in the use of ASI as a predictive tool in assessing rupture of AAA in female patients require further evaluation into other aspects of the disease, such as the ideal threshold for repair in women. Additionally, the prognostic utility of ASI is unknown where data on aneurysm expansion rates, diameter asymmetry, presence of thrombus, tortuosity, fusiform vs. saccular configuration, and adequate control of blood pressure are contributing factors and as such requires more research [35].

According to Girardi et al., an ASI <2.75 cm/m^2^ is considered low risk for rupture, while an ASI of >4.25 cm/m^2^ is considered high risk for rupture [49]. Replacing AD with ASI as a tool for predicting rupture would mean that a patient with high aneurysm diameter (e.g., 6.5 cm) and a low BSA (e.g., 2.4 m^2^) would be considered low risk for rupture. This has brought about various controversies in the utilization of ASI. It is believed that the BSA or weight varies throughout life. Therefore, any change in weight would also affect ASI calculation and hence risk estimation. It is also believed that the BSA/weight is influenced by factors that should not change the physiology of the aorta [49].

Furthermore, the aforementioned studies in favor of the use of ASI as a predictor of AAA rupture are prone to bias. Consequently, conducting a meta-analysis of these individual studies, currently lacking, would aid in bias reduction. 

Future Directions in AAA Rupture Prediction

AAA poses a significant health concern, and predicting rupture risk accurately is crucial for effective clinical management. It is a common pathology in the aging population of the developed world, carrying a significant mortality rate in excess of 80% in case of rupture. In fact, AAAs represent the only surgical condition in which size is such a critical determinant of the need for intervention. However, there are certain limitations in the prognostic value of these variables, and there is an ongoing search for additional risk markers (i.e., biomechanical parameters, morphometric characteristics, etc.).

Recent studies have proposed the importance of 3D volumetric measurements over diameter measurements in monitoring the growth rates of AAAs. A significant association with the need for surgical repair was established for AAA volumes and not for maximum diameter (D_max_) [50]. Also, a study by Wever et al. showed a 37% discordance between D_max_ and volume measurements, a decrease in aneurysm size missed using D_max_ in 14% of cases post-EVAR, and an increase in 19% of cases [51]. Vaitėnas et al, in their systematic review, also favored volume measurements in the prognosis of AAAs in 17 out of 19 studies [52].

Various biomechanical measurements including wall stress, strain, elasticity, and fluid dynamics offer a promising avenue for predicting the rupture of AAAs. Fillinger et al. demonstrated the importance of wall stress over diameter in the prediction of rupture risk by utilizing CT data, 3D computer modeling, finite element analysis, and blood pressure. Wall stress better differentiated patients who required emergent repair compared to elective repair (elective vs. emergent repair: diameter, 3% differences, P=0.5; wall stress, 38% difference, P<0.0001) [53]. Stress analysis will, therefore, be beneficial in patients with shorter life expectancy who have a wide AAA diameter but low wall stress as this will prevent complications that arise from endovascular repair. Fillinger et al. also discovered that women have a higher percentage of "high stress" aneurysms, further confirming why rupture would occur at smaller diameters compared to men [53].

Further studies have shown that the rupture potential index, RPI (ratio of locally acting wall stress to strength), is an even better predictor of AAA rupture than peak wall stress [54]. These volumetric and biomechanical techniques are yet to be put into full use in clinical practice; however, they have shown promising prospects in improving patient outcomes.

## Conclusions

Adopting the ASI as an alternative to traditional metrics like aortic diameter offers a promising avenue for improving AAA management, particularly in female patients. ASI's ability to adjust for individual body size differences provides a more accurate assessment of rupture risk, potentially leading to more equitable screening and intervention strategies. Emerging techniques such as volume measurements and biomechanical assessments hold promise in further refining rupture risk prediction and guiding personalized treatment approaches. Integrating these advancements into clinical practice can significantly improve patient outcomes and reduce the morbidity and mortality associated with AAA.
